# Impact of low-dose sevoflurane with propofol-based anesthesia on motor-evoked potentials in infants: a single-arm crossover pilot study

**DOI:** 10.1007/s00540-024-03436-z

**Published:** 2024-12-01

**Authors:** Taiki Kojima, Hirofumi Nakahari, Makoto Ikeda, Michihiro Kurimoto

**Affiliations:** 1 Department of Anesthesiology, Aichi Children’s Health and Medical Center, 7-426 Morioka-cho, Obu, Aichi 474-8710 Japan; 2 Division of Comprehensive Pediatric Medicine, Nagoya University Graduate School of Medicine, Nagoya, Japan; 3 Department of Anesthesia, St. Luke’s International Hospital, Tokyo, Japan; 4 Department of Clinical Engineering, Aichi Children’s Health and Medical Center, Obu, Japan; 5 Department of Neurosurgery, Aichi Children’s Health and Medical Center, Obu, Japan

**Keywords:** Infants, Inhalational anesthetics, Intraoperative neurophysiological monitoring, Motor-evoked potentials, Total intravenous anesthesia

## Abstract

**Purpose:**

The influence of anesthetic interactions on motor-evoked potentials in infants has rarely been reported. In infants, adding a small dose of sevoflurane to propofol-based total intravenous anesthesia is reasonable for reducing propofol administration. We collected preliminary data regarding the effect of low-dose sevoflurane in propofol-based total intravenous anesthesia on motor-evoked potentials in infants.

**Methods:**

This pilot interventional study included 10 consecutive infants requiring motor-evoked potentials between January 2023 and March 2024. The motor-evoked potential amplitudes in the upper and lower extremities were recorded twice when general anesthesia was maintained using (1) propofol-based total intravenous anesthesia and (2) 0.1–0.15 age-adjusted minimum alveolar concentration sevoflurane + propofol-based total intravenous anesthesia.

**Results:**

The motor-evoked potential amplitude in the right upper extremity was not significantly different after the addition of a small dose of sevoflurane [192 (75.3–398) μV, 121 (57.7–304) μV, *P* = 0.19]. All the motor-evoked potential amplitudes in the right lower extremity (quadriceps femoris, anterior tibialis, and gastrocnemius muscles) were significantly attenuated by adding a small dose of sevoflurane (median [interquartile range]: 47.9 [35.4–200] μV, 25.2 [12.4–55.3] μV, *P* = 0.014; 74.2 [51.9–232] μV, 31.2 [2.7–64] μV, *P* = 0.0039; 29.8 [20–194] μV, 9.9 [3.8–92.4] μV, *P* = 0.0039, respectively). Similar results were observed in the left lower extremities.

**Conclusion:**

Adding even 0.1–0.15 age-adjusted minimum alveolar concentration sevoflurane to propofol-based total intravenous anesthesia attenuated the motor-evoked potential amplitudes in the lower extremities. A further prospective interventional study with an appropriate sample size is required to investigate the study hypothesis.

**Supplementary Information:**

The online version contains supplementary material available at 10.1007/s00540-024-03436-z.

## Introduction

Motor-evoked potential (MEP) monitoring is commonly used to prevent intraoperative nerve injury. Most inhalational anesthetics are known to suppress MEP, particularly at doses > 0.5 minimum alveolar concentration (MAC) [1]. Therefore, anesthesiologists commonly choose propofol-based total intravenous anesthesia (TIVA) with opioids, which have a relatively low inhibitory effect on monitoring.

Infants are more sensitive to the suppressive effects of anesthetics on MEP because of the immaturity of their neural structures. This increases susceptibility to anesthetics, resulting in increased stimulus voltages required to elicit appreciable evoked potential amplitudes [2]. Thus, anesthesia management with propofol-based TIVA is preferable. Conversely, infants have immature liver function, and delayed propofol metabolism can cause adverse events, such as suppression of the monitoring signal amplitude (i.e., anesthetic fade) [3], delayed postoperative emergence, and propofol infusion syndrome [4]. Anesthesia management with low-dose inhalational anesthetics can limit the total intraoperative amount of propofol administered, which appears reasonable to avoid potential propofol-related complications.

Current Japanese guidelines state that co-administration of inhalational anesthetics < 0.5 MAC with different anesthetics should not affect MEP in children [5]. However, we previously reported a case of an infant in whom sevoflurane with a 0.1–0.15 age-adjusted MAC with propofol-based anesthesia significantly suppressed MEP [6]. There is limited evidence regarding the suppressive effect on MEP by the interaction between low-dose inhalational anesthetics and propofol in infants.

This pilot interventional study aimed to collect preliminary data to investigate the inhibitory effects of low-dose sevoflurane co-administered with propofol on MEP in infants.

## Materials and methods

### Ethical considerations

This study was approved by the local institutional ethics committee (Approval No. 2022058; November 1, 2022). It was registered with the Japan Registry of Clinical Trials (jRCT) (registration number: jRCT1041220109; December 17, 2022) and conducted according to the principles of the Declaration of Helsinki. Written informed consent was obtained from all patients’ guardians before inclusion.

### Study design, setting, and population

This single-arm, crossover interventional pilot study was conducted at a single tertiary care children’s hospital in Japan between January 2023 and March 2024.

Ten consecutive infants aged < 1 year without preoperative neurological complications who underwent spinal surgery and required MEP monitoring were included. Patients were excluded if (1) sevoflurane or propofol was contraindicated for use during general anesthesia, (2) hemodynamic instability defined as shock status occurred, (3) there were any signs of insufficient plane of anesthesia (e.g., body movement and detrimental airway reflexes), and (4) the patient’s guardians denied participation in this study.

### Anesthetic management

No premedication was administered to the infants. Inhalational anesthesia induction was performed with sevoflurane (inhalational concentration 5–8%) and 40% nitrous oxide with oxygen. After placing peripheral intravenous access in the upper extremity, 2.0 mg/kg of propofol and 2.0–3.0 μg/kg of fentanyl were administered via peripheral venous access. No muscle relaxants were administered during general anesthesia. Following the administration of propofol and fentanyl, nitrous oxide and sevoflurane administration was terminated, and continuous infusion of 4.0–6.0 mg/kg/h of propofol and 0.7–1.0 μg/kg/min of remifentanil was initiated for general anesthesia maintenance based on the clinical judgment of the assigned anesthesiologists. After securing the airway with an appropriately sized tracheal tube (3.0 or 3.5 mm), mechanical ventilation was initiated with 6.0 l/min of oxygen to eliminate nitrous oxide and sevoflurane. Next, the patient was placed in the prone position, and neurosurgeons and clinical engineers attached a transcranial MEP (TcMEP). At least 20 min after initiating mechanical ventilation with 6.0 l/min of oxygen, TcMEPs were recorded as control data after confirming that the end-tidal sevoflurane concentration was undetectable by the anesthesiologists assigned to the case. After recording the control data, the assigned anesthesiologists began administering 0.1–0.15 age-adjusted MAC of sevoflurane (0.25–0.35% end-tidal concentration) under mechanical ventilation with 3.0 l/min of oxygen and continuous infusion of propofol (4.0–6.0 mg/kg/h) and remifentanil (0.7–1.0 μg/kg/min). After at least 20 min of initiating sevoflurane administration, the assigned anesthesiologists confirmed that the end-tidal sevoflurane concentration was 0.1–0.15 age-adjusted MAC, followed by the second TcMEP recording (Supplemental Fig. 1). After the patient was placed in the prone position, propofol and remifentanil were maintained at the same doses until the completion of the second recording. All recordings were completed before the incision of the epidural membrane. These recordings were performed to confirm the absence of any manipulation on the infant, which could have caused noise in the recording results.

The sevoflurane dose added (0.1–0.15 MAC) was pre-determined in the study protocol prior to the data collection based on a previous case report which suggested that it could suppress TcMEP amplitudes in infants by adding it to propofol-based anesthesia [6]. In addition, the researchers (TK, HN) thoroughly discussed the impact of adding sevoflurane with propofol-based anesthesia on hemodynamical suppression while structuring a study protocol. We applied a minimal dose of sevoflurane (0.1–0.15 MAC) with propofol-based anesthesia to minimize the suppression of hemodynamic status.

### Procedure of TcMEP

An intraoperative MEP measurement system (Neuromaster-MEE1232®; Nihonkoden, Tokyo, Japan) was used to record TcMEP. Stimulating electrodes of 10 mm plated disks were placed with conductive paste at 10–20 mm anterior sites of C3 (cathode) and C4 (anode) on the International 10–20 system, assuming the motor cortex area in infants. A train-of-five square-wave pulse was delivered at an interval of 2.0 ms. The pulse duration was 50 μs. The stimulation intensity was determined at the first TcMEP recording for control data, where the intensity was set supramaximal for each stimulus (i.e., 300–600 V). The plate-shaped TcMEP detectors were placed bilaterally with conductive paste on the adductor pollicis, quadriceps femoris, tibialis anterior, and gastrocnemius muscles.

### Data collection

For data collection, electronic medical and surgical records (HAPPY ACTIS®, Canon Medical System, Tochigi, Japan) and electronic anesthetic records (Fortec ORSYS®, Phillips®, Amsterdam, The Netherlands) were reviewed to determine the patient’s underlying characteristics (month of age, sex, height, body weight, American Society of Anesthesiologists physical status, and preoperative neurological complications), and intraoperative vital signs at the moment of the first and second TcMEP recordings (i.e., blood pressure, heart rate, peripheral capillary oxygen saturation, and body temperature). TcMEPs were performed using Neuromaster-MEE1232® (Nihon Kohden, Tokyo, Japan). The voltage between the top and bottom waveforms of TcMEP amplitudes obtained from the upper and lower extremities was recorded in Neuromaster-MEE1232® (Nihon Kohden). The TcMEP recordings were performed at the adductor pollicis muscles in the upper extremities and the quadriceps femoris, anterior tibialis, and gastrocnemius muscles in the lower extremities.

### Endpoints

The primary endpoint was the change in the top-to-bottom voltage of the TcMEP in the right upper extremity between the first and second recordings. The secondary endpoint was the change in the top-to-bottom voltage of the TcMEP in the right lower extremities between the first and second recordings. Since preoperative unidentified central nervous system disturbances in the lower limbs in children could have existed owing to underlying diseases, the MEP amplitudes of the upper limbs were selected as the primary endpoint. In addition, there could have been preoperative unidentified central nervous system disturbances between the right and left sides of the children because of underlying disease. Thus, the changes in the MEP amplitudes on the left side of the upper and lower limbs were also reported as additional analysis.

### Statistical analysis

For descriptive statistics, normally and non-normally distributed continuous variables were described as means, standard deviations, medians, and interquartile ranges (IQR). Categorical variables were expressed as numbers and percentages. To evaluate the primary and secondary endpoints for paired samples, the Wilcoxon signed-rank test was used to compare the TcMEP voltages between the first and second recordings in the upper and lower extremities.

Sample size estimation was not performed during the development of the study protocol, because this study aimed to collect exploratory data. Data were analyzed using Stata V.18.0 (StataCorp, College Station, TX), with a two-tailed *P* value of < 0.05 as the criterion to decline the null hypothesis for each analysis.

## Results

### Patient characteristics and vital signs during recording

Patient characteristics are summarized in Table 1. The median (IQR) age and weight of the patients were 3.5 (3–8) months and 6.3 (5.3–7.4) kg, respectively. The doses of the anesthetics administered during the recordings are summarized in Table 2. The median value (IQR) of the total administered dosages of propofol between the initiation of the continuous infusion and the first recording was 39.1 (28.7–49.9) mg, and that between the first and second recordings was 9.6 (7.5–13.3) mg, respectively. The median value (IQR) of the total administered dosages of remifentanil between the initiation of the continuous infusion and the first recording was 257 (178–313) μg and that between the first and second recordings was 97.7 (70.6–153) μg, respectively. The median value (IQR) of the end-tidal sevoflurane concentration at the second recording was 0.30 (0.30–0.30)%. Vital signs and stimulation voltages are summarized in Table 3. The median (IQR) stimulation voltage was 500 (500–550) V.

**Table 1:** Baseline patient characteristics (*n* = 10)

ID	Sex	Age (months)	Weight (kg)	Diagnosis	PMA at birth (weeks)	Preoperative hemoglobin (g/dL)
1	Female	5	7.1	Spinal lipoma	39	11.4
2	Female	3	5.3	Spinal lipoma	39	13.2
3	Female	8	8.0	Spinal lipoma	41	11.9
4	Female	10	7.4	Spinal dermoid cyst	37	9.8
5	Male	3	6.0	Spinal lipoma	38	10.7
6	Male	9	8.9	Spinal lipoma	39	12.0
7	Female	2	4.9	Spinal lipoma	37	12.2
8	Male	2	4.6	Spinal lipoma	36	10.9
9	Male	4	6.6	Spinal lipoma	39	11.4
10	Female	3	5.8	Spinal lipoma	38	11.6

*PMA* postmenstrual age

The median (interquartile range) age and weight of the patients were 3.5 (3, 8) months and 6.3 (5.3, 7.4) kg, respectively

Propofol and remifentanil dosages were the same for the first and second recordings in all cases

**Table 2:** Doses of administered anesthetics (*n* = 10)

ID	Propofol dose (mg)		Remifentanil dose (mcg)		End-tidal sevoflurane concentration at the second recording (%)
	Before 1st recording	Between 1st and 2nd recording	Before 1st recording	Between 1st and 2nd recording	
1	43.7	13.3	177.5	152.7	0.40
2	28.7	9.2	148.4	119.8	0.20
3	49.9	11.2	424	168	0.30
4	36.7	22.2	313.4	333	0.30
5	59	8.8	313.2	105.6	0.40
6	75.8	13.5	279	67.5	0.30
7	35.7	7.4	240	70.6	0.30
8	41.4	6.1	251	51	0.30
9	21.1	7.5	253	89.8	0.30
10	28.2	10	60.3	80	0.30

The median value (IQR) of the total administered dosages of propofol between the initiation of the continuous infusion and the first recording was 39.1 (28.7, 49.9) mg, and that of between the first and second recordings was 9.6 (7.5, 13.3) mg, respectively

The median value (IQR) of the total administered dosages of remifentanil between the initiation of the continuous infusion and the first recording was 257 (178, 313) mcg, and that of between the first and second recordings was 97.7 (70.6, 153) mcg, respectively

The median value (IQR) of the end-tidal sevoflurane concentration at the second recordings was 0.30 (0.30, 0.30)%

**Table 3:** Vital signs and stimulation voltages in first and second recordings (*n* = 10)

ID	Mean blood pressure (mmHg)		Peripheral arterial oxygenation saturation (%)		Heart rate (bpm)		End-tidal carbon dioxide concentration (mmHg)		Nasopharyngeal temperature (℃)		Stimulation voltage (V)
	1st	2nd	1st	2nd	1st	2nd	1st	2nd	1st	2nd	
1	61	65	100	100	107	125	34	34	35.8	35.8	600
2	54	55	99	98	81	85	38	36	36.7	36.6	600
3	52	54	100	100	112	121	40	40	36.7	36.8	500
4	67	71	100	100	91	88	35	38	36.8	36.6	500
5	59	56	100	100	115	113	37	37	36.0	35.9	500
6	57	44	97	99	105	111	39	38	36.9	36.8	450
7	45	46	100	100	105	103	36	36	36.1	36.0	500
8	43	43	100	100	101	109	42	39	37.1	37.0	500
9	52	52	100	100	97	99	37	37	36.3	36.3	550
10	49	46	100	100	115	118	35	36	36.2	36.3	480

The stimulation voltages were the same for the first and second recordings in all the cases

The median (IQR) stimulation voltage was 500 (500, 550) V

#### Primary endpoint

The MEP amplitude in the right upper extremity (adductor pollicis muscle) was not significantly different after the addition of a small dose of sevoflurane (median [IQR]: 192 [75.3–398] μV, 121 [57.7–304] μV, *P* = 0.19) (Table 4).

**Table 4:** Amplitudes of motor-evoked potentials (μV) in the right upper and lower extremities (*n* = 10)

ID	Adductor pollicis muscles		Quadriceps femoris muscles		Tibialis anterior muscles		Gastrocnemius muscles	
	1st	2nd	1st	2nd	1st	2nd	1st	2nd
1	200	84.1	44.9	31.7	15.3	2.7	20	2.7
2	732	317	355	205	595	26.3	772	116
3	72.8	57.7	31.8	27.3	81.2	64	89.2	5.3
4	75.3	65.8	19.1	12.4	67.1	37.4	22.4	13.3
5	690	159	49	0	0	0	0	0
6	398	304	200	150	191	187	194	118
7	13.9	11.4	239	23	273	36	34.9	17.8
8	266	491	46.8	5.8	51.9	9.4	24.7	3.8
9	185	287	35.4	55.3	232	64.2	197	92.4
10	105	16.9	133	16.4	64.4	0	16.6	6.4

Zero indicates the absence of a response to motor-evoked potential stimulation

#### Secondary endpoint

All the MEP amplitudes in the right lower extremity (quadriceps femoris, anterior tibialis, and gastrocnemius muscles) were significantly attenuated by adding a small dose of sevoflurane (median [IQR]: 47.9 [35.4–200] μV, 25.2 [12.4–55.3] μV, *P* = 0.014; 74.2 [51.9–232] μV, 31.2 [2.7–64] μV, *P* = 0.0039; 29.8 [20–194] μV, 9.9 [3.8–92.4] μV, *P* = 0.0039, respectively) (Table 4, Supplemental Fig. 2).

### MEP amplitudes in the left upper and lower extremities

The MEP amplitude in the left upper extremity (adductor pollicis muscle) was not significantly different after the addition of a small dose of sevoflurane (median [IQR]: 193 [160–464] μV, 114 [31.0–315] μV, *P* = 0.33). All the MEP amplitudes in the left lower extremity (quadriceps femoris, anterior tibialis, and gastrocnemius muscles) were significantly attenuated by adding a small dose of sevoflurane (median [IQR]: 148 [48.5–298] μV, 30.8 [17.6–77.2] μV, *P* = 0.013; 107 [19.0–326] μV, 18.5 [14–43.8] μV, *P* = 0.0051; 144 [20.4–239] μV, 19.1 [6.3–103] μV, *P* = 0.0051, respectively) (Table 5, Supplemental Fig. 3).

**Table 5:** Amplitudes of motor-evoked potentials (μV) in the left upper and lower extremities (*n* = 10)

ID	Adductor pollicis muscles		Quadriceps femoris muscles		Tibialis anterior muscles		Gastrocnemius muscles	
	1st	2nd	1st	2nd	1st	2nd	1st	2nd
1	176	53.6	48.5	14.4	19	16.7	19	5.9
2	720	36.2	298	30.8	326	18.6	269	6.3
3	11.6	9	33.3	17.6	136	116	200	157
4	200	559	6.8	19.6	17.4	10.7	7	5.6
5	560	175	66	30.7	14	0	39	21.9
6	464	328	261	70.4	77	40.6	187	103
7	13.9	15.1	230	0	155	14.0	101	59
8	160	315	63.6	77.2	37.2	18.3	20.4	10
9	200	31	444	275	400	43.8	243	16.3
10	186	277	594	226	676	665	239	208

Zero indicates the absence of a response to motor-evoked potential stimulation

## Discussion

This pilot interventional study confirmed the feasibility of conducting a prospective interventional study to evaluate the effect of the interaction between propofol-based TIVA and a small dose of sevoflurane on the MEP amplitude. In addition, the current exploratory data showed that adding a small dose of sevoflurane to propofol-based TIVA attenuated MEP amplitudes in the lower extremities, even with a limited sample size.

Halogenated anesthetic agents, including sevoflurane, increase gamma-aminobutyric acid type A and glycine receptor activity, downregulating the activity of cholinergic and NMDA-type glutamate receptors and suppressing neural activities by inhibiting voltage-gated potassium and sodium channels [7]. Thus, propofol-based TIVA with remifentanil, which has a minimal inhibitory impact on MEP, is commonly used for general anesthesia maintenance for surgeries applying MEP [8, 9]. Specifically, for neonates and infants, propofol-based TIVA with remifentanil seems optimal when applying MEP in these populations, because children < 3 years old have a high threshold of obtaining proper MEP response owing to the immature central nervous system where a larger voltage and higher pulse stimulation are required to obtain a proper MEP response [10]. However, intraoperative propofol accumulation can occur in infants, especially those < 6 months, by prolonged propofol infusion because of reduced clearance [11]. This may cause several problems, including delayed emergence, dyslipidemia, propofol infusion syndrome, and metabolic acidosis [11]. Therefore, co-administration of a small dose of inhalational agents (e.g., sevoflurane) to minimize the propofol infusion dose is reasonable (e.g., sevoflurane, remifentanil).

Previous studies supported the availability of sevoflurane usage to obtain an acceptable range of MEP amplitudes in infants [12–14]. Moreover, current Japanese guidelines suggest co-administering < 0.5 age-adjusted MAC inhalational agents with low-dose propofol can be allowed regarding the inhibitory impact of MEP [5]. However, there is a lack of evidence regarding the impact of the interaction between inhalational anesthetics and propofol anesthesia on MEP response in infants. Moreover, Adynlar et al. reported that a high MEP stimulating voltage was needed to record acceptable MEP response under general anesthesia with sevoflurane [12]. Previously, we reported a case of an infant where MEP amplitudes disappeared with reproducibility by adding 0.1–0.15 age-adjusted MAC sevoflurane on propofol-based TIVA (66.6 μg/kg/min of propofol and 0.50 μg/kg/min of remifentanil) [6]. This case report found a similar suppressive tendency of additional low-dose sevoflurane on propofol-based TIVA. Therefore, no conclusive evidence exists regarding the impact of adding low-dose sevoflurane on propofol-based TIVA on MEP response in neonates and infants.

Our findings also showed a contradictory inhibitory effect of sevoflurane on the MEP amplitude in the upper and lower extremities. There are several potential explanations. First, the stimulating electrode may have been placed closer to the primary motor cortex of the upper extremities. The area for the movement of the lower extremities is located close to the midline, while that of the upper extremities is located on the lateral side [15], which could cause contradictory responses in the upper and lower extremities. Second, infants’ immature development of nervous system structures could be related to attenuating action potential conduction on nerves. In children, myelination and synaptogenesis in the nervous system develop during the first 2–3 years of life, when the nerve conduction system and neural signaling at nerve junctions are immature [16]. These anatomical and physiological immaturities require a larger threshold of MEP voltage to obtain a proper response while increasing the susceptibility to a small dose of sevoflurane, attenuating the MEP amplitudes. This may occur especially in longer nerve pathways from the primary cortex to the peripheral effector cells (likely to occur more in the lower extremities than in the upper extremities). More nerves or nerve junctions can be exposed to the MEP inhibitory effect of sevoflurane in the longer nerve conductance pathways, resulting in significant MEP voltage suppression in the lower extremities. Finally, there could have been unapparent nerve disturbances due to underlying disease. This micro-nerve injury may have interacted with the influence of adding a low dose of sevoflurane. However, this pilot study included a minimal sample size, leading to an underpowered analysis. Thus, further investigation with appropriate sample size is required to evaluate the MEP suppression in the upper extremities caused by adding a small dose of sevoflurane.

There are several important limitations in this preliminary study. First, propofol accumulation could cause the suppression of MEP amplitudes in the second recording (i.e., anesthetic fade). Therefore, further interventional studies must compare cases where propofol and remifentanil without sevoflurane are used for anesthesia maintenance [17]. Second, the methodology of measuring MEPs in infants has not been adequately established. This can lead to false-positive results of MEP monitoring (MEP response is absent even without nerve injury) that can cause a measurement bias. In this study, we carefully adjusted the location of stimulating electrodes and the intensity and frequency of the MEP stimulation. However, evidence regarding the optimal methodology for conducting MEP monitoring in infants is lacking. Further studies are required to establish stable MEP monitoring in this pediatric population. Finally, the sample size in this study was small because of the nature of the preliminary study. However, this study can provide benchmark data to facilitate the optimal sample size assumption. Thus, further interventional studies might be able to utilize our data for sample size estimation.

Conclusively, this pilot study in infants showed the feasibility of conducting a prospective interventional study to evaluate the effect of adding a small dose of sevoflurane to propofol-based TIVA on MEP amplitude. In addition, the preliminary data showed the suppressive effect of adding a small dose of sevoflurane to propofol-based TIVA on MEP amplitude, even in a limited sample size. However, a prospective study with an appropriate sample size is required to confirm the current results.

## Supplementary Information

Below is the link to the electronic supplementary material.

Supplementary file1 (TIF 90 KB)

Supplementary file2 Motor-evoked potential amplitudes in the right upper and lower extremities between the first and second recordings are described. A: Adductor pollicis muscle, B: Quadriceps femoris muscle, C: Anterior tibialis muscle, D: Gastrocnemius muscle. 1st: 1st recording, 2nd: 2nd recording. (TIF 66 KB)

Supplementary file3 Motor-evoked potential amplitudes in the left upper and lower extremities between the first and second recordings are described. A: Adductor pollicis muscle, B: Quadriceps femoris muscle, C: Anterior tibialis muscle, D: Gastrocnemius muscle. 1st: 1st recording, 2nd: 2nd recording. (TIF 71 KB)

## Data Availability

The anonymized data that support the findings of this study can be provided by the principal investigator upon reasonable request.

## Declarations

### Prior presentation

Parts of these findings were reported during an invited presentation at the 71st Annual Meeting of the Japanese Society of Anesthesiologists in Kobe, Japan on June 6, 2024.
