# Supplementary figures and images for: Impact of low-dose sevoflurane with propofol-based anesthesia on motor-evoked potentials in infants: a single-arm crossover pilot study
Source: J Anesth. 2024 Dec 1;39(1):93–100. doi: 10.1007/s00540-024-03436-z (PMC11782304; doi:10.1007/s00540-024-03436-z)

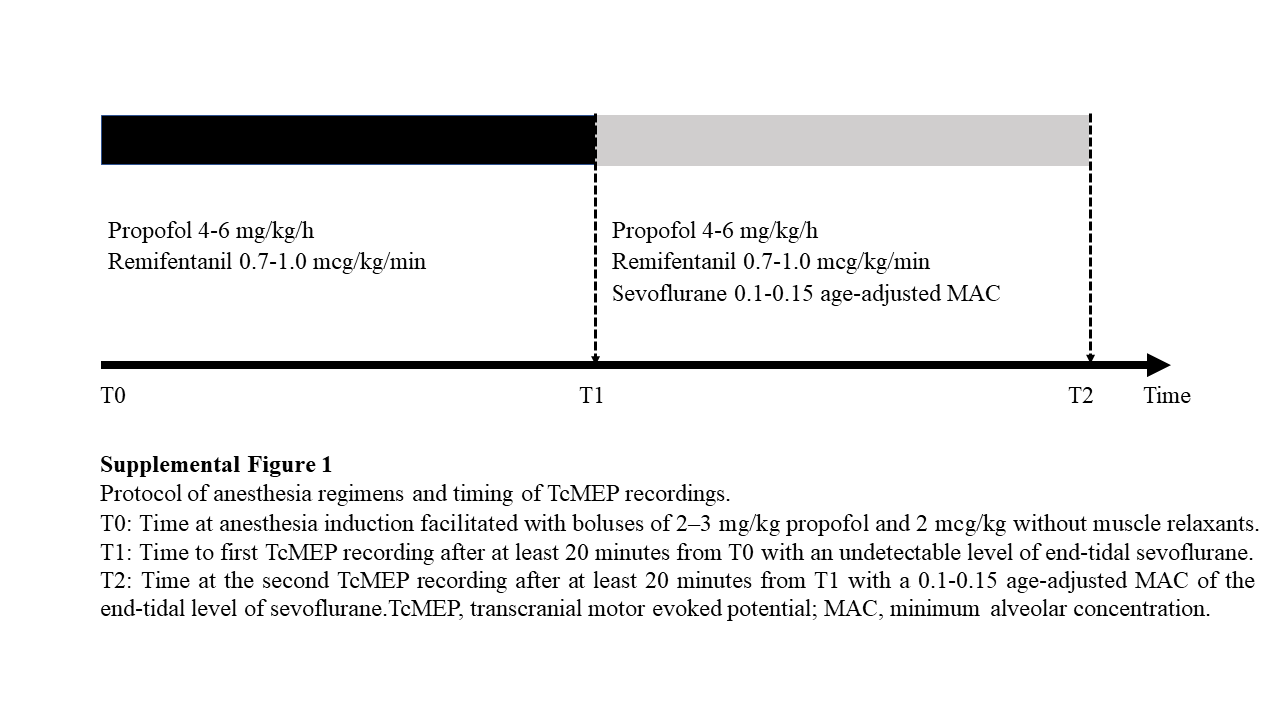

Supplement: Supplementary file 1 — Supplementary file1 (TIF 90 KB) [file 540_2024_3436_MOESM1_ESM.tif]

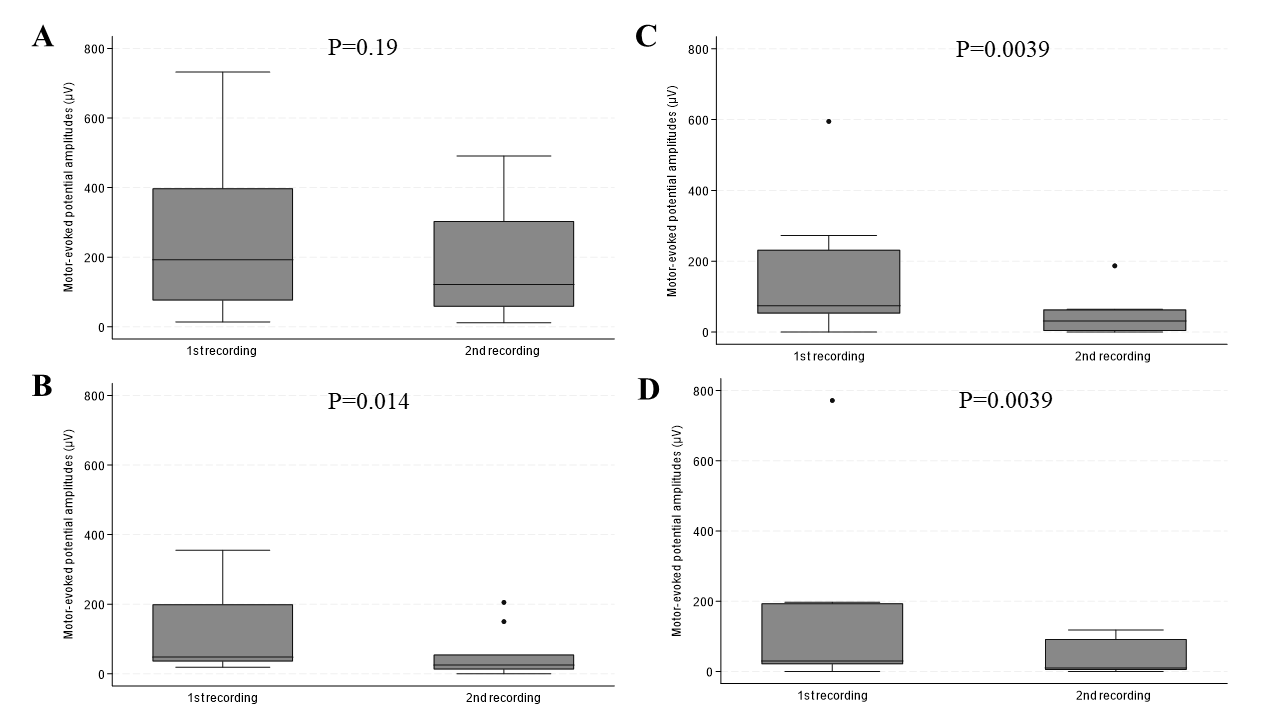

Supplement: Supplementary file 2 — Supplementary file2 Motor-evoked potential amplitudes in the right upper and lower extremities between the first and second recordings are described. A: Adductor pollicis muscle, B: Quadriceps femoris muscle, C: Anterior tibialis muscle, D: Gastrocnemius muscle. 1st: 1st recording, 2nd: 2nd recording. (TIF 66 KB) [file 540_2024_3436_MOESM2_ESM.tif]

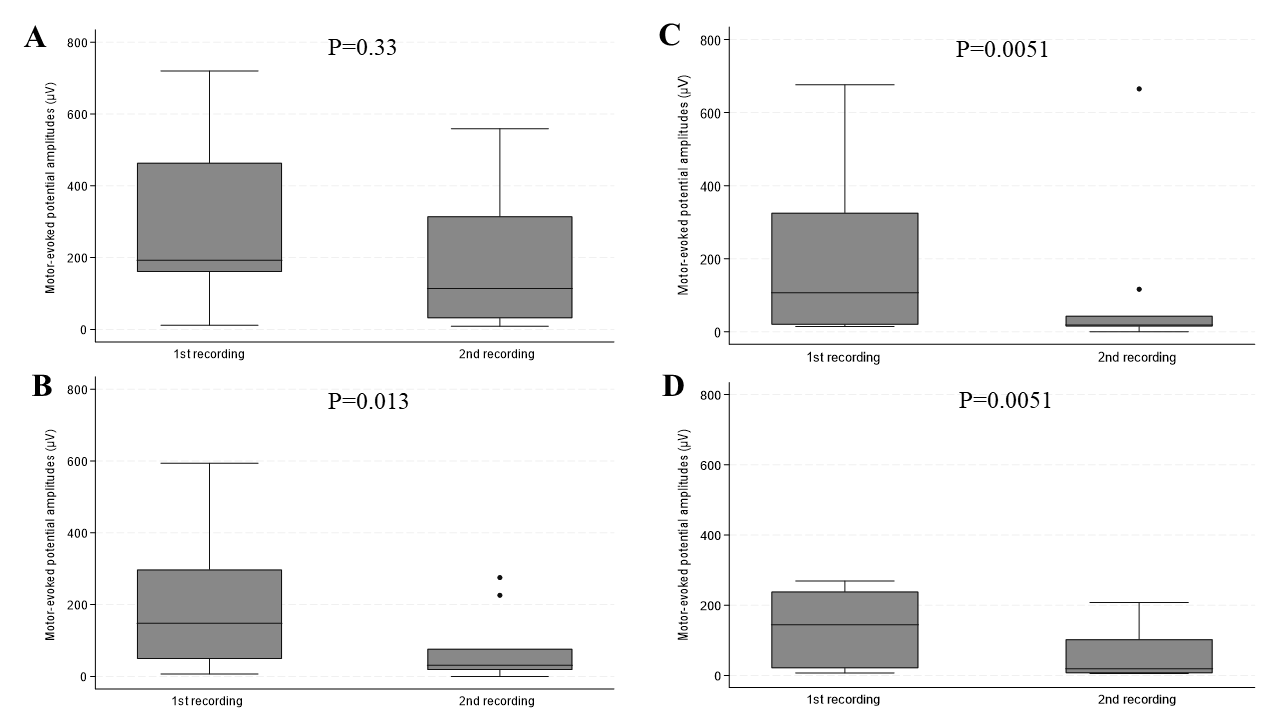

Supplement: Supplementary file 3 — Supplementary file3 Motor-evoked potential amplitudes in the left upper and lower extremities between the first and second recordings are described. A: Adductor pollicis muscle, B: Quadriceps femoris muscle, C: Anterior tibialis muscle, D: Gastrocnemius muscle. 1st: 1st recording, 2nd: 2nd recording. (TIF 71 KB) [file 540_2024_3436_MOESM3_ESM.tif]
